# Mutational landscape of intestinal crypt cells after long-term in vivo exposure to high fat diet

**DOI:** 10.1038/s41598-023-41123-3

**Published:** 2023-08-26

**Authors:** Mathilde Meyenberg, Anna Hakobyan, Nikolina Papac-Milicevic, Laura Göderle, Franziska L. Langner, Mateo Markovic, Ji-Hyun Lee, Bon-Kyoung Koo, Georg A. Busslinger, Israel Tojal da Silva, Christoph J. Binder, Jörg Menche, Joanna I. Loizou

**Affiliations:** 1 CeMM Research Center for Molecular Medicine of the Austrian Academy of Sciences, 1090 Vienna, Austria; 2 Center for Cancer Research, Comprehensive Cancer Center, Medical University of Vienna, 1090 Vienna, Austria; 3 Department of Structural and Computational Biology, Max Perutz Labs, University of Vienna, 1030 Vienna, Austria; 4 Department of Laboratory Medicine, Medical University of Vienna, 1090 Vienna, Austria; 5 Division of Gastroenterology and Hepatology, Department of Internal Medicine III, Medical University of Vienna, 1090 Vienna, Austria; 6 Institute of Molecular Biotechnology of the Austrian Academy of Sciences (IMBA), Vienna BioCenter (VBC), Dr. Bohr-Gasse 3, 1030 Vienna, Austria; 7 Center for Genome Engineering, Institute for Basic Science, 55, Expo-Ro, Yuseong-Gu, Daejeon, 34126 Republic of Korea; 8 Laboratory of Computational Biology and Bioinformatics, A.C. Camargo Cancer Center, São Paulo, 01508-010 Brazil; 9 Faculty of Mathematics, University of Vienna, 1090 Vienna, Austria

**Keywords:** Intestinal stem cells, Computational biology and bioinformatics, Risk factors, Cancer genetics, Cancer prevention, Gastrointestinal cancer, DNA damage and repair

## Abstract

Obesity is a modifiable risk factor in cancer development, especially for gastrointestinal cancer. While the etiology of colorectal cancer is well characterized by the adenoma-carcinoma sequence, it remains unclear how obesity influences colorectal cancer development. Dietary components of a high fat diet along with obesity have been shown to modulate the cancer risk by perturbing the homeostasis of intestinal stem cells, yet how adiposity impacts the development of genomic instability has not been studied. Mutational signatures are a powerful way to understand how a complex biological response impacts genomic stability. We utilized a mouse model of diet-induced obesity to study the mutational landscape of intestinal crypt cells after a 48-week exposure to an experimental high fat diet in vivo*.* By clonally enriching single crypt derived cells in organoid culture and obtaining whole genome sequences, we analyzed and compared the mutational landscape of intestinal epithelial cells from normal diet and high fat diet mice. Single nucleotide substitution signatures and indel signatures present in our cohort are found equally active in both diet groups and reflect biological processes of normal aging, cellular replication, and oxidative stress induced during organoid culturing. Thus, we demonstrate that in the absence of activating mutations or chemical exposure, high fat diet alone is not sufficient to increase genomic instability.

## Introduction

Global obesity rates have been steadily increasing for the past 40 years^1^. Obesity is accompanied by many comorbidities such as increased likelihood of type II diabetes, hypertension, and nonalcoholic fatty liver disease^1, 2^. Among the biggest health impacts is the increase in cancer risk which accompanies body fat accumulation^3–6^. The International Agency of Research on Cancer (IARC) has recognized the overwhelming epidemiological evidence which links the chronic obese condition with increased cancer risk, in particular for organs along the gastro-intestinal axis^7^. Especially the risk of developing colorectal cancer (CRC) is highly influenced by dietary risk factors and high body mass index (BMI)^8^. With the clear association between high BMI and CRC risk, gaining understanding of the underpinning disease etiology could inform preventative as well as therapeutic programs.

Colorectal cancer development is defined by a well described progression of mutations, known as the adenoma-carcinoma sequence^9^. Deactivating mutations in adenomatous polyposis coli (*APC*) are initiating mutations, leading to constitutive Wnt/β-catenin signaling. Colorectal cancer develops through three different molecular pathways, the chromosomal instability pathway (CIN), the microsatellite instability pathway (MSI), and the CpG island methylation pathway (CIMP)^10^. Although the development of CRC is heterogeneous and sometimes involves overlapping pathways, all three pathways are defined by genomic instability which enables the acquisition of further mutations in a set of tumor suppressor and oncogenes, including *KRAS* and *BRAF* (often mutually exclusive), *TP53, PIK3CA*, and *SMAD4*^10, 11^. Interestingly, it was shown that concomitant loss of APC and p53 is sufficient to induce high levels of chromosomal instability, characteristic for the CIN pathway^12^. Despite well-defined molecular genetics in CRC development, it remains unclear how a high fat diet (HFD) impacts this series of events.

With the advent of advanced tissue culturing techniques, it has become possible to study the most relevant cell populations in vitro^13^. In the case of CRC, the cell population of origin are the rapidly cycling LGR5 positive (leucine rich repeat containing G protein coupled receptor 5) intestinal stem cells (ISCs), residing at the bottom of the crypt^14^. These cells have been demonstrated to be sensitive to dietary and metabolic perturbation, modulating the risk of cancer initiation^15–18^. A prolonged exposure to HFD constituents has been shown to confer stemness features on non-stem cell progenitors, thus increasing the pool of actively replicating cells^16, 19^. The HFD component palmitic acid was found to initiate this effect via the activation of *PPAR-∂* (peroxisome proliferator-activated receptor delta) signaling, which induces canonical Wnt-signaling^16, 19^. Another prominent metabolite commonly associated with diet induced obesity is cholesterol. Extended exposure to high cholesterol levels were found to also drive proliferation of ISCs and increase the rate of tumorigenesis in an *APC* deficient background^17^.

Although it has been demonstrated that a HFD directly modulates signaling in the stem cell niche, the effect on genomic stability has not been studied yet. Beyond describing mutations in individual genes, mutational signatures offer a framework to systematically study how genomic instability arises in cancer development. Mutational signatures are a mathematical framework that allows to define patterns of mutations within their sequence context. The specific mutational imprint of a signature on the genome is the reflection of the dysregulation or dysfunction of DNA damage and repair pathways and other biological processes^20^. Since the conception of mutational signatures in 2013^21, 22^, it has become possible to investigate cancer genomes at a global level and capture patterns which describe complex underlying biological mechanisms. Bottom-up in vitro studies, measuring the mutagenic effect of an exposure or gene knockout, have proven to be especially useful in defining signature etiologies^23–26^.

Here, we investigated whether exposure to prolonged high fat diet generates distinct mutational processes in intestinal crypt cells. Because mutational signatures effectively capture biologically complex processes, they serve as a good readout for studying effects on genomic stability. We sequenced and analyzed clonal intestinal organoids derived from mice which were fed an experimental HFD for 48 weeks. After data processing and variant calling, we obtained sufficient numbers of single base substitution (SBS) and indel (ID) mutations to investigate SBS and ID signatures, as well as coding mutations. For both diet groups, we recover expected signatures related to aging, tissue culture processing, and cellular replication. We demonstrate that differential mutagenesis is not initiated by HFD alone in the absence of other disturbance events, such as chemical exposure or mutations in CRC driver genes.

## Results

### Mouse model of dietary induced obesity

**Figure 1:**
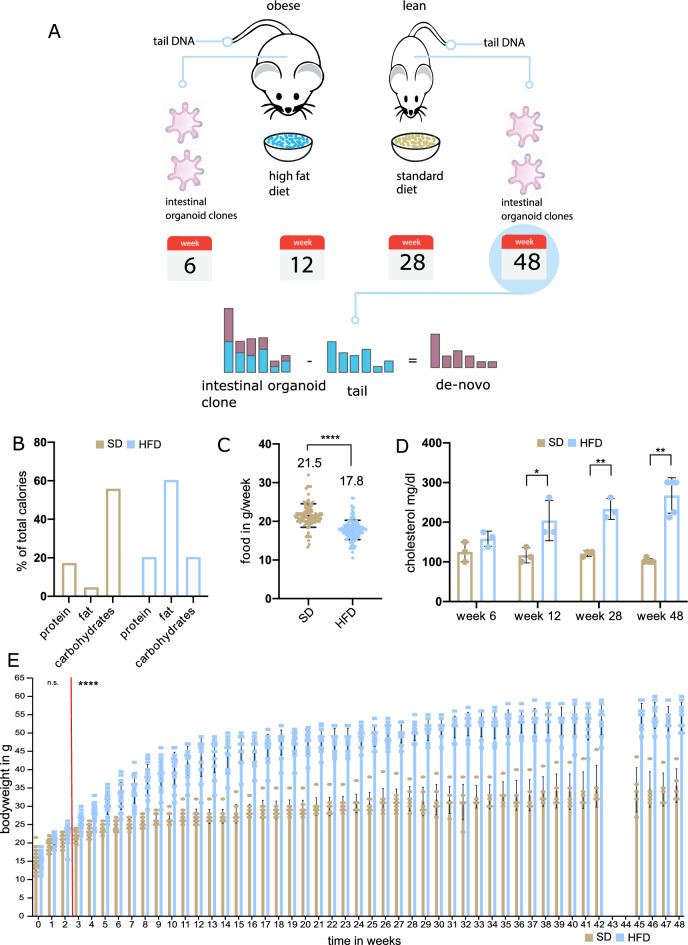
(**A**) Schematic display of experimental workflow. (**B**) Macronutrients of experimental diets shown by percent contribution to total calories. HFD is shown in light blue and SD is shown in light brown. (**C**) Food consumption per diet group, measured per cage and divided by the number of mice per cage. The group average and statistical significance is indicated above (unpaired t-test, two-tailed) (**D**) Plasma cholesterol content in mg/dl shown per diet group at each point of the time course. N = 3 for each group at timepoints week 6, 12, 28 and N = 5 at week 48 (pairwise t-test, two-tailed). (**E**) Weekly weight measurements for diet groups. Dots indicate measurements for individual mice. Statistically significant weight gain was observed after 3 weeks on the HFD (indicated by red line) Statistical significance was tested using multiple unpaired t-tests with alpha = 0.001 (Holm–Sidak correction method for multiple testing, not assuming consistent standard deviation between groups).

To study the long-term effect of obesity on genomic stability in the intestinal crypt, we set up a cohort of age matched male C57/BL6J mice (Fig. 1A). After random assignment to cages with either standard chow (SD) or HFD, the mice were started on the respective diet course at the age of 5 weeks for 48 continuous weeks. At set time intervals of 6, 12, 28, and 48 weeks, a random subsample of HFD and SD mice was drawn and sacrificed to harvest ISCs for culturing. Organoids were picked and cultured to clonality before obtaining whole genome sequences (30×) for 5 obese and 5 lean mice from the last timepoint (48 weeks). For each mouse, 4 independent organoid clones and the matched tail were sequenced to distinguish acquired variants from germline variants. The clonal organoid lines take on a cystic morphology characteristic of intestinal organoids with high stem cell content^13^ and are positive for Lgr5 expression (Supplementary Fig. 1A, B).

Our model of diet induced obesity relies on the choice of supplied diet. In the high fat diet condition, mice derive 60% of all calories from fat, while the majority of calories in the normal diet (SD) derive from carbohydrates (55.5%) (Fig. 1B). The exact diet composition is described in Supplementary Tables 1 and 2. Despite lower overall food consumption in the HFD group (Fig. 1C), mean weekly caloric intake was higher in the HFD group (92.7 kcal/week) compared to the SD group (80.2 kcal/week). C57BL/6J mice have been well characterized as model organisms for diet induced obesity, capturing essential aspects of metabolic dysregulation and weight gain^27, 28^. Our cohort also exhibited the marked increase in total cholesterol upon exposure to HFD (Fig. 1D) and a significant increase in body weight after 3 weeks on the HFD (Fig. 1E), recapitulating the metabolic dysregulations and resulting phenotype associated with obesity.

### Qualitative analysis of mutational profiles in SD and HFD fed mice

**Figure 2:**
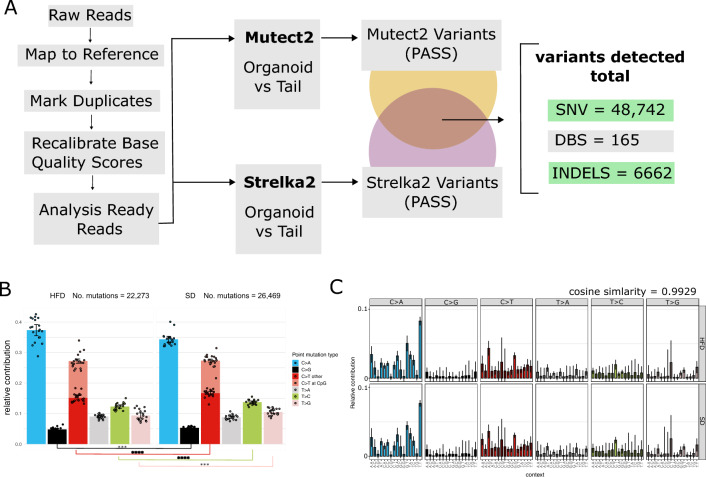
(**A**) Raw reads from paired end 150 bp Illumina sequencing were processed according to GATK best practices, including marking and removal of duplicates and recalibration of base quality scores. Analysis ready reads were processed by two mutation callers, Mutect2 and Strelka2. Variants called by both tools were included in the analysis. In total, 48,742 single nucleotide variants, 165 double base substitution variants, and 6662 insertions and deletions could be detected. (**B**) Relative contribution of SNVs in six mutation classes for HFD samples (left panel) and SD samples (right panel). C > T mutations within CpG sites are shown as a separate category. Individual dots indicate organoid samples, error bars show ± 1 sd from the mean, asterisks indicate results from pairwise t-test (two-sided) comparing mutation numbers for each mutation category, alpha = 0.05 (**C**) Average mutational profile of SNVs in 96 channels shown for HFD (upper panel) and SD (lower panel). Error bars indicate ± 1 sd.

Since the genome records past and ongoing mutational processes, we reasoned that longer exposure to HFD would result in a stronger signal. Hence, we focused our sequencing efforts on the last time point (48 weeks). The obtained raw reads were processed according to GATK (The Genome Analysis Toolkit) best practices (Fig. 2A). To obtain a high confidence set of mutations, we utilized two mutation callers, Mutect2^29, 30^ and Strelka2^31^. Mutations which were found by both Mutect2 and Strelka2 and passed the respective quality filter settings were included for further analysis. This yielded a total of 48,742 single nucleotide variants (SNV), 165 double nucleotide substitutions (DSB), and 6662 indels (insertions and deletions). Due to low numbers of mutations, DSBs were excluded from further analysis. As an additional quality control step, we checked the variant allele frequency (VAF) distribution of all organoid clones and included only clonal samples, where the VAF distribution is centered around 0.5 (Supplementary Fig. 1C).

We first explored the overall mutational landscape for SNVs per diet group. Surprisingly, we found a slightly higher number of total mutations in the SD group than in the HFD group. We observed a significantly higher number of mutations in the SD group for C > G, C > T outside of CpG regions, T > C, and T > G (Fig. 2B). The profile of relative contributions, across the 7 mutation channels, however, is similar between the two diet groups. Next, we examined the mutational profiles in 96 channels. The mean mutational profile per diet group exhibits few characteristic peaks, with the exception in the C > A and C > T components. The aggregated profile of the HFD group has a cosine similarity of 0.9929 to the SD group (Fig. 2C). We furthermore observe highly similar profiles between mice of either diet group (Supplementary Fig. 2A,B). To quantify how similar the mutational profiles of samples across diet groups are, we computed the pairwise cosine similarity between all samples, which ranges from 0.9020 to 0.9776 (mean = 0.9558) (Supplementary Fig. 2C).

The high cosine similarity between all samples implies the absence of strong differential mutational processes. To test this, we used a bootstrap resampling method of the 96-channel mutation matrix, adapted from Zou et al*.*, for SD and HFD samples^24^. This allows us to detect potential qualitative differences in mutational profiles which remain uncovered due to low sample size and high signal to noise ratio. The global bootstrapped mutational profile of the SD mice has a cosine similarity of 0.9933 when compared to the profile of the HFD mice (Supplementary Fig. 2D).

In summary, this suggests that no strong qualitative differences exist for mutagenic processes in either diet group.

### Mutational signature analysis of single nucleotide variant profiles

#### De-novo signature extraction of SNV signatures

Despite the lack of qualitative differences in the mutational profiles of the two diet groups, we next sought to explore which mutational signatures are active in the diet groups to determine whether quantitative differences exist. We first employed non-negative matrix factorization (NMF) with automated rank selection based on the NMFk method to determine the optimal number of de-novo signatures to extract^32, 33^. Classically, NMF algorithms use heuristics to determine the optimal rank based on either stability of the solution, or on automatic relevance determination (ARD), which is a measure of precision of the chosen model to explain the data^33^. In contrast, the NMFk method for automatic rank determination seeks to optimize the tradeoff between both, the stability of the solution and the accuracy of the reconstructed data, measured as the distance between original and reconstructed profiles (mean sample cosine distance). This method allows to robustly extract a meaningful number of signatures from noisy data while minimizing the number of false positive signatures^32^.

**Figure 3:**
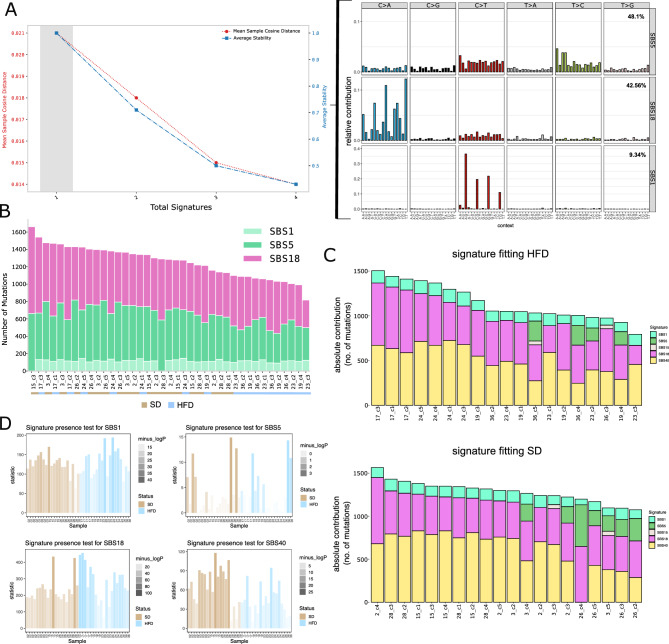
(**A**) NMF for signature extraction ranging from 1 to 4 signatures. Red line indicates mean sample cosine distance (MSCD), blue line indicates average stability (AS), gray bar indicates preferred solution, maximizing the tradeoff between MSCD and AS. The decomposition of the extracted signature into known COSMIC signatures and their calculated percent contribution is shown to the right. (**B**) NMF results from A shown as per sample absolute signature contributions (number of mutations), diet status of the samples is indicated at the bottom. (**C**) Best subset signature refitting using signatures commonly active in colorectal cancer. Per sample absolute signature contributions (number of mutations) are shown for HFD samples (upper panel) and SD samples (lower panel). (**D**) Signature Presence test for 4 most active signatures. The y-axis indicates the likelihood ratio between the signature fitting with and without the tested signature. The translucence of the bars, shown for individual organoid clones, is indicative of the level of significance (-log p).

Applied to our data, both stability and mean sample cosine distance decline the more signatures are extracted. Thus, the most optimal solution consists of extracting a single signature (Fig. 3A). The presence of a single consensus profile in the cohort would indicate that there are no distinguishing signatures between diet groups. The de-novo extracted signature can furthermore be decomposed into known signatures from the catalog of somatic mutations in cancer (COSMIC) database^34^. According to this decomposition, the *de-novo* signature consists of 48.1% SBS5, 42.56% SBS18, and 9.34% SBS1 (Fig. 3A). Comparing the per sample contribution of the decomposed signatures reveals an equal distribution of signature activities across samples, regardless of diet used (Fig. 3B).

#### Signature refitting of SNV signatures

In cohorts with lower sample numbers such as ours, an alternative approach to de-novo extraction is signature refitting, where the mutational catalog of the samples is fitted to the catalog of known signatures (COSMIC) to find a subset which best explains the observed mutational catalog. This approach takes a defined set of known signatures and performs a refit in an iterative manner. After each iteration the reconstructed and original profile are compared and the lowest-contributing signature is eliminated from the set. Signatures will stop being removed when the cosine similarity between the reconstructed and original profile between two iterations has changed more than a given threshold. Thus, only signatures which are necessary to model the observed data are retained in the set. By repeating this process n − 1 times for all sets of n − 1, n − 2, n − 3 etc., where n is the total number of known signatures, we find that SBS1, SBS5, SBS18, and SBS40 explain 99.6% of observed mutations in both diet groups (Fig. 3C). Only four samples showed minimal activity of SBS15 (defective mismatch repair), a signature directly attributable to an increase in genomic instability. The equally minimal number of mutations attributed to SBS15 in both diet groups, however, suggest no differential potential in mismatch repair among SD and HFD. In summary, the distribution of the fitted signatures is highly similar across samples and is not diet specific.

To quantify the activity of the most active signatures, we applied the signature presence test from the mSigAct package^35, 36^ to SBS1, SBS5, SBS18, and SBS40. This statistical test builds two refit models, one including and one excluding the signature of interest, while minimizing the reconstruction error. Following this, the likelihood ratio test between the two models is computed. For ratios greater than 1, the likelihood of the signature being active is significantly higher than the alternative hypothesis. The signature presence test confirmed the results obtained from signature refitting. Of the 4 tested signatures, SBS5 is the least active, as already observed before (Fig. 3D). Although some variation in signature activity can be observed between individual samples, no signature shows a systematic difference between diets.

All signatures we found are equally active in both diet groups and are likely attributable to normal aging processes and the culturing process prior to sequencing. SBS1 is a clock-like signature which is attributed to aging due to spontaneous deamination of 5-methylcytosines, which leads to a C > T transition^21^. The activity of SBS1 observed in both groups thus likely reflects the normal aging process. Additionally, both groups showed high numbers of C > A mutations, which were largely attributed to SBS18. This signature has been proposed to be caused by damage due to reactive oxygen species^22, 25^ and might thus have arisen during the routine experimental handling of the samples or due to exposure to metabolic byproducts in the intestine. The remaining signatures SBS5 and SBS40 share similarly flat profiles. Although only SBS5 has been clearly identified as a clock-like signature, SBS40 was also found to correlate with age^22, 37^. Thus, the activity of both signatures may be explained by normal aging processes. Taken together, the results from de-novo extraction and signature refitting, confirm that the experimental HFD did not induce or impact different mutational processes for single nucleotide substitutions compared to the standard diet.

### Mutational signature analysis of indel profiles

#### Comparison of indel profiles between diet groups

**Figure 4:**
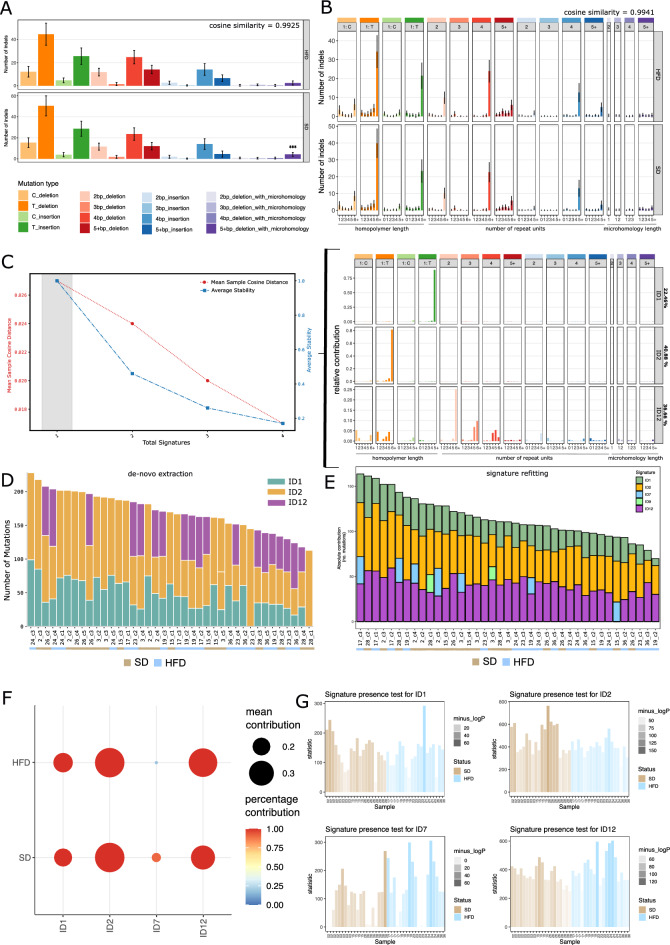
(**A**) Main indel context (16-channels) profiles aggregated by diet group (mean), error bars indicate ± 1 sd. Statistical significance was assessed using multiple pairwise t-tests, not assuming consistent standard deviation (Holm–Sidak correction method for multiple testing, alpha = 0.01) (**B**) Mean extended context indel profile by diet group (83 channels), error bars indicate ± 1 sd from the mean. (**C**) NMF diagnostic plot for signature extraction ranging from 1 to 4 signatures. Red line indicates mean sample cosine distance (MSCD), blue line indicates average stability (AS), gray bar indicates preferred solution, maximizing the tradeoff between MSCD and AS. The decomposition of the extracted signature into known COSMIC indel signatures and their calculated percent contribution is shown to the right. (**D**) NMF results from A shown as per sample absolute signature contributions (number of mutations), the diet status of the samples is indicated on the x-axis. (**E**) Best subset signature refitting using all 18 known indel signatures. Per sample absolute signature contributions (number of mutations) are shown. Diet status is indicated on the x-axis. (**F**) Bootstrapped refitting of indel signatures (best subset approach using all known indel signatures, 100 iterations). Size of dots indicates the mean contribution of the signature for all bootstrap iterations where this signature was found. The color scale represents the percentage of bootstrap iterations where the signature was found active. (**G**) Signature Presence test for 4 most active signatures found in refitting and bootstrapped refitting. The x-axis indicates the sample, the y-axis indicates the likelihood ratio between the signature fitting with and without the tested signature. The translucence of the bars, shown for individual organoid clones, is indicative of the level of significance (− log p).

Aside from SNVs, numerous mutational processes also generate insertions and deletions. This class of mutations generates signatures different to SNV signatures. We therefore analyzed the 6662 indel mutations in our cohort to compare whether differences in indel generating mutational processes exist between the diet groups. We only considered clonal samples with a VAF distribution centered around 0.5 (Supplementary Fig. 3). Indel mutations can be analyzed in 16 or in 83 curated channels, representing the main and extended sequence context respectively^38^. The curated indel types range from a single base pair deletion or insertion, up to indels longer than 5 bp. Additionally, 1–5 bp deletions flanked by microhomologies are considered, since such mutations are indicative of defective double strand break repair processes^39^. Indel profiles in both sequence contexts were highly similar between diet groups, with a cosine similarity of 0.9925 for main indel contexts (Fig. 4A), and 0.9941 for extended indel contexts (Fig. 4B). Only 5 + bp deletions flanked by microhomologies were significantly increased in the SD compared to the HFD cohort (Fig. 4A). However, since the total number of mutations in that category is less than 10, this likely represents a random variation and carries no specific biological meaning. Indeed, all mice, regardless of diet group, exhibited highly similar indel profiles, both for the main and the extended sequence context (Supplementary Fig. 4A–D). Furthermore, all samples showed a pairwise cosine similarity greater than 0.84 (Supplementary Fig. 4E). Conclusively, the high cosine similarity between indel profiles of the diet groups as well as among individual samples suggest that no indel generating processes are unique to either diet.

#### De-novo signature extraction of indel signatures

We next applied the same analysis workflow we established for SNV signatures to all insertions and deletions. NMF with automated rank selection, found one indel signature as the optimal solution because extraction of more than one signature led to a sharp decrease in average stability (Fig. 4C left panel). The decomposition of the single de-novo signature estimated three known COSMIC signatures to be active, ID1 (22.46%), ID2 (40.88%), and ID12 (36.66%) (Fig. 4C right panel). The distribution of the signature contribution to the individual samples does not differ between diet groups (Fig. 4D).

#### Signature refitting for indel signatures

Exploring indel signatures further with refitting analysis allowed us to confirm the results obtained from de-novo extraction. Using best subset refitting with all 18 known indel signatures, we find ID1, ID2, and ID12 most active and similarly distributed across samples (Fig. 4E). Minor activity observed for ID7 (MMR deficiency^37^) and ID9 (etiology unknown^37^), may be due to signature misattribution for the common C and T deletions found in our cohort. Since the low number of mutations may be limiting in this analysis, we also pooled the mutational matrix of each diet group and performed a best subset refit to all COSMIC indel signatures. The results show an equal distribution of ID1, ID2, and ID12 activity across diet groups (Supplementary Fig. 4F). To confirm the stability of the refitting, we performed bootstrapped refitting. The mutational matrix is resampled 1000 times with replacement, using the original mutational profile as weight. For each bootstrap iteration a refit is calculated, recording the estimated signature activity. The higher the consensus of refits across bootstrap iterations, the more stable the refit. The results confirm that ID1, ID2, and ID12 are the most active signatures in our cohort, regardless of diet consumed. ID7 was found active only in the SD group and was attributed less than 10% of all mutations in that group (Fig. 4F). Finally, we quantified the signature activity of ID1, ID2, ID7, and ID12 for all samples, using the signature presence test (Fig. 4G). The results confirm that ID2, and ID12 (etiology unknown^37^) are the most active signatures in both diet groups, since the majority of mutations is attributed to these signatures across all samples. ID1 is the third most active indel signature, followed by ID7, which is active only in some samples and completely absent in 23% of all samples.

None of the identified indel signatures are differentially active between tested diets. Signatures ID1 and ID2 are both proposed to arise due to slippage of the replicated (ID1), and template strand (ID2) during replication, producing the characteristic 5 + bp T-insertions and 6 + bp T-deletions. These signatures have been observed to be active in all samples and are only increased in backgrounds with mismatch repair deficiency (MMR)^37^. In our cohort, we have not observed a strong activity of either SNV or indel signatures associated with defective mismatch repair. The low activity of MMR deficiency signature ID7 in some samples may partially explain the high activity observed for ID1 and ID2. However, this process is equally active in both diet groups (Fig. 4E,G). Notably, ID1 and ID2 activity were found increased in conditions of chronic inflammation of the intestinal tract in patients^40^. Even though obesity is associated with changes in metabolic and hormonal signaling associated with forming an inflammatory environment^6^, we do not observe an increase in ID1 and ID2 activity that would indicate strong changes in inflammatory signaling. Thus, the activity of ID1 and ID2 we find in both diet groups suggest mutational processes ongoing during normal cellular replication. In summary, the results indicate that the experimental HFD did not invoke or influence mutational processes of indel generation.

### Coding mutations

**Figure 5:**
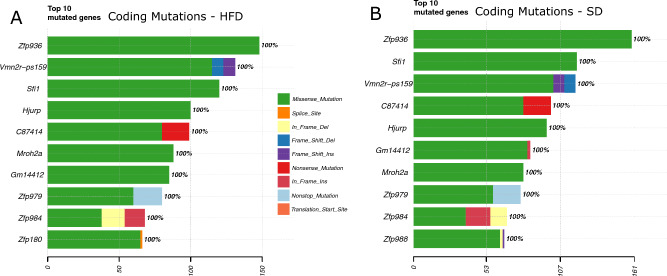
(**A**) Top 10 coding mutations in HFD (**B**) and SD mice. The type of substitution is indicated by color. The percentage of samples with a mutation in the gene is indicated to the right.

Finally, we wondered whether the absence of specific mutational processes also precluded the accumulation of specific deleterious mutations which might initiate the adenoma-carcinoma sequence and thus predispose to tumor development. To test this, we explored the coding mutations which accumulated in either diet group. Due to low numbers of coding mutations in our cohort, we included all mutations which passed the filtering criteria from the Mutect2 variant caller. Filtering for the most mutated genes revealed a remarkable overlap, 9 out of the top 10 most mutated genes are shared between the SD and HFD group (Fig. 5A,B). The largest fraction of alterations are missense mutations. None of the mutated genes have a known role in intestinal cancer development. Taken together, we found no specific mutations which might explain how obesity increases risk of cancer development in the intestinal tract.

## Discussion

Obesity is a chronic disease which epidemiologically has been shown to increase the risk of developing cancer in the intestinal tract^3–7^. High BMI and dietary factors such as consumption of a western style diet high in fats have been demonstrated to have a positive association with CRC risk through modulation of signaling in the intestinal stem cell niche^15–19^. However, the effect of obesity on the genomic stability of intestinal stem cells has not been investigated yet. We hypothesized that chronic exposure to a HFD impacts on DNA damage and repair signaling or associated processes and thus shapes the landscape of genomic stability in intestinal stem cells. To investigate the mutational landscape in response to diet induced obesity we used whole genome sequencing on clonal organoid populations derived from intestinal crypt cells of mice exposed to experimental high fat or control diets, respectively.

Our results show that HFD alone, on an isogenic background, and in the absence of other predisposing mutations, does not induce differential mutational signatures compared to a standard control diet. All mutational signatures we recovered are equally active in both diet groups and represent normal ongoing mutational processes associated with aging (SBS1, SBS5), cellular replication (ID1, ID2), or oxidative stress experienced either in-vivo or during the culturing process during sample preparation (SBS18). Overall, signatures we recover are in agreement with previous findings, reporting the activity of SBS1, 5, and 18, as well as ID1 and ID2 as normal aging and metabolism associated processes in colonic crypts^40^. Other signatures recovered were SBS40 and ID12, which are both signatures with unknown etiology and were active in both diet groups. We furthermore found no coding mutations in common CRC driver genes or other genes associated with developing genomic instability. Thus, in the absence of any other predisposing mutations, diet-induced obesity associated alterations to any molecular pathways in the stem cell niche are not enough to generate an excess of mutations, specific mutational patterns or coding mutations that would predispose to cancer. The lack of mutagenesis in the HFD condition, both in terms of numbers of mutations and mutational signatures, would suggest that the DNA repair machinery is working efficiently in the diet-induced obesity condition, ensuring genomic stability. While we did not profile epigenetic changes in this study, it is known that HFD induces epigenetic remodeling of the enhancer landscape in murine colonic epithelial cells, activating enhancers of CRC-associated genes^41, 42^. Prolonged exposure to HFD eventually leads to transcriptional reprogramming, downregulating tumor-suppressors, while at the same time upregulating genes associated with enhanced proliferation, without initiating tumors in the absence of further stressors^43^. Considering our results in the context of previous studies, this would indicate that both, epigenetic changes and the regulation of the DNA damage and repair machinery change in concert in response to HFD and prime the intestinal epithelium toward cancer initiation. How a HFD impacts on the mutagenesis in a cancer predisposed background (e.g. ApcMin) or in the presence of DNA damaging agents remains to be investigated.

In the face of convincing epidemiological evidence, it remains important to understand how a western style diet modulates cancer risk in the gastrointestinal tract. By investigating mutagenesis in intestinal crypt cells upon long-term exposure to a high fat diet in vivo, we show that HFD alone, in the absence of other perturbation events, such as mutations or chemical exposure, is not sufficient to initiate specific mutational patterns. Our results might lead to future studies to investigate combinatorial effects of HFD with other perturbations, to continue elucidating the etiology of obesity induced cancers.

## Methods

### Mouse work

All experimental protocols were approved by the institutional animal experimentation committee of the Medical University of Vienna and the Austrian Ministry of Science under ethical permit number 66.009/0179-V/3b/2019. All methods were performed in accordance with relevant guidelines and regulations. The study was reported in accordance with the ARRIVE guidelines. Experimental mice were age-matched males on a C57BL/6J background. The total number of mice included the study was 28. Control and treatment groups (diet groups) were randomly assigned cage numbers before the addition of the experimental research diets. Researchers were not blinded to the assigned treatment.

Shortly, from 5 weeks of age, after a 1-week acclimatization period on SD, mice were fed SD or HFD for 6, 12, 28, and 48 weeks (Research diets, D12492i, rodent diet with 60 kcal% fat, for diet composition see Supplementary Tables 1, 2). Mice were housed at the Department of Laboratory Animal Science and Genetics of the Medical University of Vienna, Austria with a 12-h dark-/light-cycle with ad libitum access to water and food. Weight gain and food consumption of experimental animals were monitored on a weekly basis. At experimental exitus mice were sacrificed after 3 h of fasting. Blood, plasma, intestinal tissues, and intestinal crypts from the jejunum for organoid culture were isolated. Blood was collected from the vena cava with a syringe, stored in collection tubes containing EDTA, and spun down to 15 min at 2000*g*. The supernatant plasma was retrieved and snap frozen in liquid nitrogen.

### Organoid culture

Isolated tissue from jejunum was gently rinsed with ice cold PBS (20 mL, without Mg++ and Ca++) using a syringe. The intestinal tube was cut lengthwise and covered with fresh PBS (1–2 mL, without Mg++ and Ca++)). The villi were gently scraped off using a microscope coverslip. Following this, the tissue was cut into ca. 0.5 cm long pieces and added to a tube containing ice cold PBS (50 mL, without Mg++ and Ca++). The tissue pieces were washed by gently inverting the tube before collecting the tissue pieces and repeating this washing process 2 more times with fresh PBS. After washing, tissue pieces were collected and incubated in enzyme-free dissociation buffer (StemCell Catalog #100-0485) for 10 min at room temperature on a tube roller. After incubation, the tube was vigorously shaken to loosen the crypts from the remaining tissue. The resulting solution was filtered through a 70 µm cell strainer and centrifuged for 3 min at 1200 rpm. Supernatant was discarded and the pellet was resuspended in fresh PBS (1 mL). Multiple aliquots of 50 µL, 100 µL, and 200 µL were transferred to 1.5 mL tubes and centrifuged for 5 min at 500 rcf. The supernatant was removed carefully and Matrigel (20 µL) was added and mixed with the pellet. The mixture was plated into 48-well tissue culture plates (20 µL per well), the plate inverted and incubated for 5 min at 37 °C to allow the Matrigel to polymerize. Finally, the droplets were covered with 250–300 µL of WENR culturing medium (Advanced DMEM/F12, 1%Glutamax(200 mM), 1% HEPES (1 M), 1% Penicillin/Streptomycin, 2% B27(50x, Thermo Fischer Catalog #17504044), 0.25% *n*-acetyl-l-cysteine (500 mM), 0.05% Recombinant Murine EGF (500 µg/mL), 0.1% Recombinant Murine Noggin (100 µg/mL, Peprotech Catalog #250-38), 0.2% Primocin (50 mg/mL, InVivoGen Catalog #ant-pm-05), 0.01% Y-27632 (100 mM, Adooq Bioscience Catalog #A11001-50), 1% Nicotinamide (1 M), 50% Wnt3A conditioned medium as described previously, 10% R-spondin conditioned medium prepared as described previously).

After 5–7 days in culture, organoids were recovered from Matrigel and dissociated into single cells using 0.05% Trypsin–EDTA (incubation at 37 °C for 5–12 min) and mechanical disruption via vigorous pipetting. Single cells were plated in increasingly diluted aliquots and checked under the microscope for complete dispersion. Resulting clonal organoids were picked with a pipette after 7–10 days in culture (medium change every 2–3 days), disrupted with 0.05% Trypsin–EDTA, and cultured until enough material was available for DNA extraction.

### Western blotting

Organoids were harvested in cold DMEM/F12, pelleted by centrifugation at 500 rcf for 5 min and digested with1x TrypLE for 5 min at 37 °C. After digestion, organoids were washed with cold PBS and pelleted by centrifugation at 500 rcf for 5 min. Cell pellets were resuspended in 50 µL RIPA buffer (150 mM NaCl, 1%Triton X-100, 0.5% sodium deoxycholate, 0.1% SDS, 50 mL TRIS pH 8.0), snap-frozen in liquid nitrogen and stored at – 20 °C until further analysis. Protein concentration of 1:3 and 1:10 dilutions were determined by BCA assay (#23227, Thermo Scientific) in duplicates according to manufacturer’s instructions. 10 µg of protein was diluted in RIPA buffer to a total volume of 15 µL (12 µL + 3 µL 5xLane marker reducing sample buffer (#39000, Thermo Scientific)). The samples were heated to 95 °C for 5 min and loaded onto a 4–15% precast polyacrylamide gel (#4568086, BioRad) alongside the Prestained Protein Ladder (PageRuler™ 10 to 180 kDa, #26616, Thermo Scientific). The SDS-page was run in 1× running buffer at 100 V for 5 min and subsequently at 85 V for 1.5 h (10× running buffer: 0.25 M TRIS, 1.924 M Glycine, 0.03467 M SDS). The nitrocellulose membrane was activated in Methanol for 5 min, washed in 1 × transfer buffer and semi-dry transfer was performed at 22 V for 22 min (10 × transfer buffer: 1.924 M Glycine, 0.25 M TRIS, 10% MeOH). The blot was shortly washed in PBS-T, blocked for 1 h in 5% milk PBS-T at RT and incubated with primary antibody in 10 mL 5% milk in PBS-T over night at 4 °C (Lgr5: ab75850, abcam 1:1000). The next day, the blot was washed 3 × 5 min in PBS-T, incubated with secondary antibody (#7074S, Cell Signaling 1:10,000) in 10 mL 5% milk in PBS-T for 1 h at RT and washed 3 × 5 min in PBS-T. Detection was performed using the SuperSignal West Femto Maximum Sensitivity Substrate (#34095, Thermo Scientific). Chemiluminescence was recorded using a Chemidoc XRS + system (BioRad). After detection, the blot was shortly washed in PBS-T, stripped in 50 mL stripping buffer (#21059, Thermo Scientific) at 37 °C, washed in PBS-T, re-blocked for 1 h in 5% milk PBS-T at RT and re-probed as described above (Gapdh: sc-32233, SantaCruz 1:8000, secondary: #7076S, Cell Signaling 1:10,000).

### Whole genome sequencing and variant calling

Organoids were extracted from the Matrigel by adding protease K (800 U, ~ 20 µg), centrifuging the solution at 500 rcf for 5 min, and discarding the supernatant. Total DNA (~ 1 µg/sample) was extracted using a QIAamp DNA Micro Kit (Qiagen Catalog #56304). Library preparation (350 bp inserts) and sequencing (150 bp PE) on a NovaSeq6000 platform (Illumina) was carried out with Novogene, Cambridge, UK. Raw reads were processed according to GATK4 best practices recommendation for data pre-processing for variant discovery. Reads were mapped to the mm10/GRCm38 mouse reference genome. All bam files were downsampled to match the file with the lowest coverage using the DownsampleSam command from Picard tools with accuracy = 0.001. Variants in organoid clones were called with Mutect2 and Strelka2, using the tail DNA as a reference. Variants with filter status PASS which were called by both tools were included in the analysis. For each sample, the variant allele frequency (VAF) distribution was plotted. All samples which did not have a distribution centered around 0.5 were excluded from further analysis.

### Mutational signature analysis

De-novo mutational signature extraction using NMF was performed using SigprofilerExtractor^32^. Signature refitting and plotting was performed using the MutationalPatterns package in R^44^. The bootstrapping analysis of the SNV signatures was conducted as described previously^24^. Briefly, bootstrapped resampling was applied to generate 10,000 replicates of the mutational matrix for SD samples and HFD samples respectively, using the underlying distribution of signatures across the 96 channels as weight. The results were aggregated by diet group to generate an average bootstrapped mutational profile, which was then compared between groups using cosine similarity.

### Supplementary Information

Supplementary Information.

## Data Availability

All code used to analyze the data and produce the figures is available on https://github.com/menchelab/hfd-mutagenesis.
