# Supplementary material for: Mutational landscape of intestinal crypt cells after long-term in vivo exposure to high fat diet
Source: Sci Rep. 2023 Aug 26;13:13964. doi: 10.1038/s41598-023-41123-3 (PMC10460443; doi:10.1038/s41598-023-41123-3)
Supplement: Supplementary file 1 — Supplementary Information. [file 41598_2023_41123_MOESM1_ESM.pdf]

Mutational Landscape of Intestinal Crypt Cells After Long-term In Vivo Exposure to High Fat Diet

Mathilde Meyenberg[1,2,3 +], Anna Hakobyan[1,3, +], Nikolina Papac-Milicevic[4], Laura Göderle[4], Franziska Langner[1,5], Mateo Markovic[4], Ji-Hyun Lee[6,7], Bon-Kyoung Koo[6,7], Georg A. Busslinger[1,5], Israel Tojal da Silva[8], Christoph J. Binder[4], Jörg Menche[1,3,9]*, and Joanna I. Loizou[1,2*]

[1]CeMM Research Center for Molecular Medicine of the Austrian Academy of Sciences, Vienna, 1090, Austria
[2]Center for Cancer Research, Comprehensive Cancer Centre, Medical University of Vienna, 1090 Vienna, Austria [3]Department of Structural and Computational Biology, Max Perutz Labs, University of Vienna, 1030 Vienna, Austria
[4]Department of Laboratory Medicine, Medical University of Vienna, Vienna, 1090, Austria
[5] Department of Internal Medicine III, Division of Gastroenterology & Hepatology, Medical University of Vienna, Vienna, 1090, Austria
[6]Institute of Molecular Biotechnology of the Austrian Academy of Sciences (IMBA), Vienna BioCenter (VBC), Dr. Bohr-Gasse 3, 1030 Vienna, Austria.
[7]Center for Genome Engineering, Institute for Basic Science, 55, Expo-ro, Yuseong-gu, Daejeon, 34126, Republic of Korea
[8]Laboratory of Computational Biology and Bioinformatics, A.C. Camargo Cancer Center, São Paulo, 01508-010, Brazil
[9]Faculty of Mathematics, University of Vienna, 1090 Vienna, Austria
*joerg.menche@univie.ac.at
*joanna_loizou@hotmail.com
+these authors contributed equally to this work

Supplementary Material

Supplementary Table 1. Composition of the experimental control diet (SD).

Control Diet (Normal Diet)
Total energy density: 3.73 kcal/g

Carbo-hydrates	g/kg	Fatty Acids	g/kg	Protein	g/kg	Vitamins	Unit per kg	Minerals	g/kg	Other	g/kg
N-free extract	550	C16:0	5.0	Arginine	8.0	Vitamin A	15,000 IU	Calcium	0.01	Ash	70
		C18:0	2.0	Cysteine	3.5	Vitamin D3	1,200 IU	Phosphorus	0.0065	Fiber	43
		C20:0	0.1	Histidine	4.0	Vitamin E	0.09	Sodium	0.003		
		C18:1	9	Isoleucine	6.5	Vitamin K	0.005	Magnesium	0.0025		
		C18:2	19	Leucine	17.0	Thiamine (B1)	0.015	Iron	0.2		
		C18:3	7.5	Lysine	8.0	Riboflavin (B2)	0.01	Iodine	0.004		
				Methionine	4.0	Pyridoxine (B6)	0.01	Copper	0.015		
				Phenylalanine	8.5	Cobalamine (B12)	0.05	Cobalt	0.0015		
				Threonine	6.0	Biotin	0.2	Manganese	0.12		
				Tryptophan	2.0	Choline	1.0	Selenium	0.0002		
				Tyrosine	6.0	Folate	0.002	Zinc	0.075		
						Niacin	0.04				
						Pantothenic Acid (B5)	0.02				

Supplier: http://www.lasvendi.com/en/lasqcdiets-eng/mice-rats/rod16-r-eng.html
Data sheet: https://www.lasvendi.com/files/PDF-EN/lasqcdiet_rod16_rad_data_eng.pdf

Supplementary Table 2. Composition of the experimental high fat diet (HFD).

High Fat Diet
Total energy density: 5.21 kcal/g

Carbo-hydrates	g/kg	Fatty Acids	g/kg	Protein	g/kg	Vitamins	g/kg	Minerals	g/kg	Other	g/kg
Lodex 10	161.53	Soybeann Oil (USP)	32.31	Casein (Lactic), 30 Mesh	258.45	Choline Bitartrate	2.58	Potassium Citrate (Monohydrate)	213.22	Solka Floc, FCC200 (Fiber)	64.61
Sucrose	94.0	Lard	316.6	L-Cysteine	3.88	Vitamin E Acetate (50%)	6.46	Calcium Phosphate (Dibasic)	167.99	Blue FD&C #1, Aluminium Lake 35-42% (Dye)	0.065
						Niacin (B3)	1.94	Calcium Carbonate (light, USP)	71.07		
						Biotin (1%)	1.3	Sodium Chloride	33.47		
						Pantothenic Acid (B5)	1.03	Magnesium Sulfate (Heptahydrate)	33.29		
						Vitamin D3 (100,00 IU/gm)	0.65	Magnesium Oxide (Heavy, DC USP)	5.41		
						Vitamin B12 (0.1% Mannitol)	0.65	Ferric Citrate	2.71		
						Vitamin A Acetate (500,000 IU/gm)	0.52	Manganese Carbonate Hydrate	1.58		
						Pyridoxine HCl (B6)	0.45	Zinc Carbonate	0.72		
						Riboflavin (B2)	0.39	Chromium Potassium Sulfate	0.25		
						Thiamine HCl (B1)	0.39	Copper Carbonate	0.16		
						Folic Acid	0.13	Ammonium Molybdate Tetrahydrate	0.039		
						Menadione Sodium Bisulfite	0.054	Sodium Fluoride	0.029		
								Sodium Selenite	0.0065		
								Potassium Iodate	0.0065		

Supplier: https://researchdiets.com/formulas/d12492
Data sheet: https://researchdiets.com/formulas/d12492

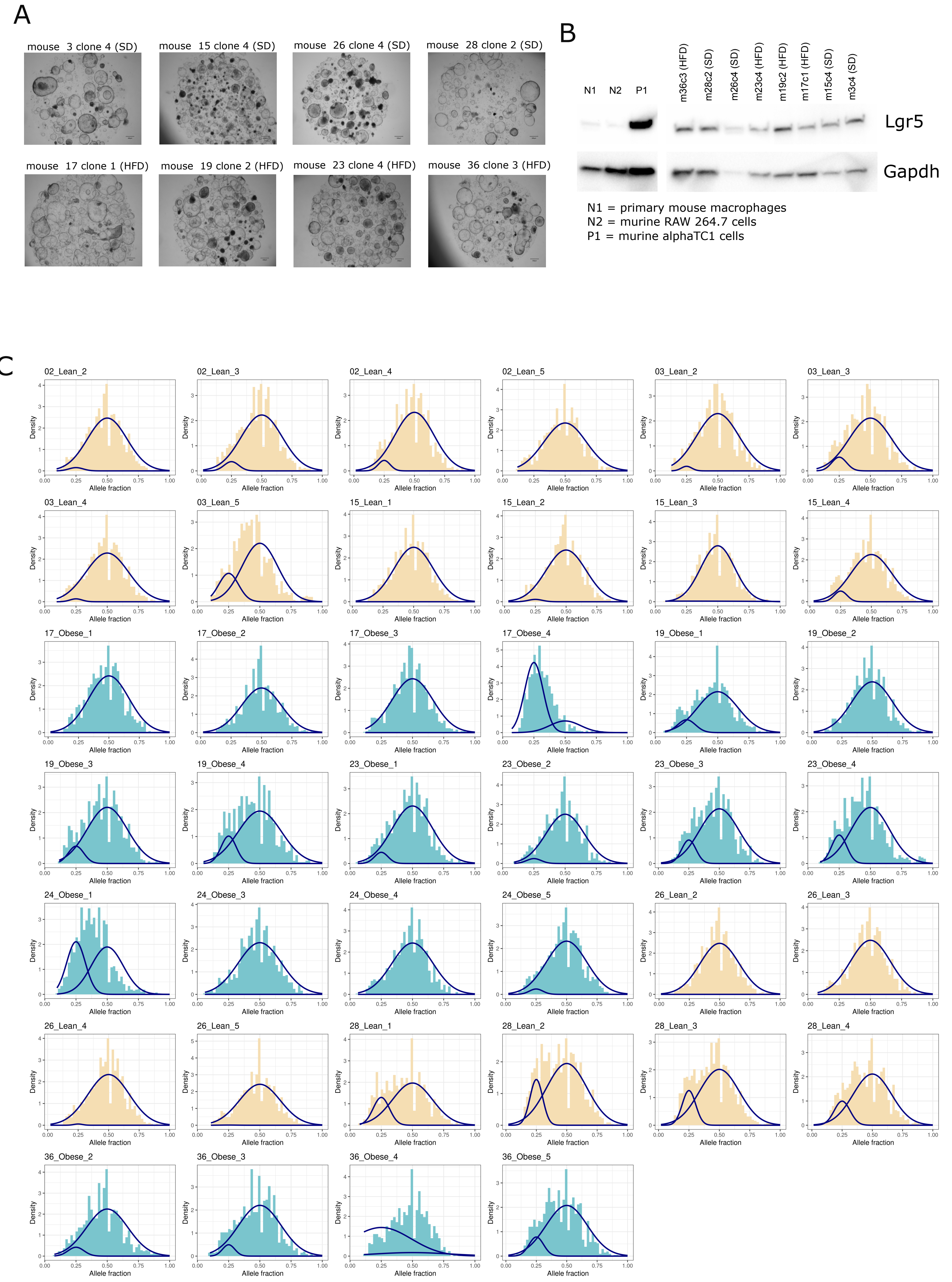
A
mouse 3 clone 4 (SD)
mouse 15 clone 4 (SD)
mouse 26 clone 4 (SD)
mouse 28 clone 2 (SD)
mouse 17 clone 1 (HFD)
mouse 19 clone 2 (HFD)
mouse 23 clone 4 (HFD)
mouse 36 clone 3 (HFD)
B
N1
N2
P1
m36c3 (HFD)
m28c2 (SD)
m26c4 (SD)
m23c4 (HFD)
m19c2 (HFD)
m17c1 (HFD)
m15c4 (SD)
m3c4 (SD)
Lgr5
Gapdh
N1 = primary mouse macrophages
N2 = murine RAW 264.7 cells
P1 = murine alphaTC1 cells
C
02_Lean_2
02_Lean_3
02_Lean_4
02_Lean_5
03_Lean_2
03_Lean_3
03_Lean_4
03_Lean_5
15_Lean_1
15_Lean_2
15_Lean_3
15_Lean_4
17_Obese_1
17_Obese_2
17_Obese_3
17_Obese_4
19_Obese_1
19_Obese_2
19_Obese_3
19_Obese_4
23_Obese_1
23_Obese_2
23_Obese_3
23_Obese_4
24_Obese_1
24_Obese_3
24_Obese_4
24_Obese_5
26_Lean_2
26_Lean_3
26_Lean_4
26_Lean_5
28_Lean_1
28_Lean_2
28_Lean_3
28_Lean_4
36_Obese_2
36_Obese_3
36_Obese_4
36_Obese_5
Density
Allele fraction

Supplementary Figure 1. (A) Representative Images of a selection of the sequenced organoid clones **(B)** Western blot showing the expression of Lgr5 in a selection of clonal organoid lines. Negative controls = N1 (primary mouse macrophages) and N2 (murine RAW 264.7 macrophages, Abelson murine leukemia virus-induced tumor) Positive control = P1 (murine alphaTC1 cells, pancreatic adenoma) Loading control = Gapdh. Uncropped gels are shown in Supplementary Figure 5. **(C)** Variant allele frequency distribution (VAF) of single nucleotide variants (SNVs) for each organoid clone modeled with a Gaussian distribution, after deduction of germline variants found in the mouse tail sequences. Gaussian mixture model was fit with fixed means at 0.25 and 0.5 to identify the proportions of clonal and sub- clonal cell populations. The resulting distributions are shown in blue.

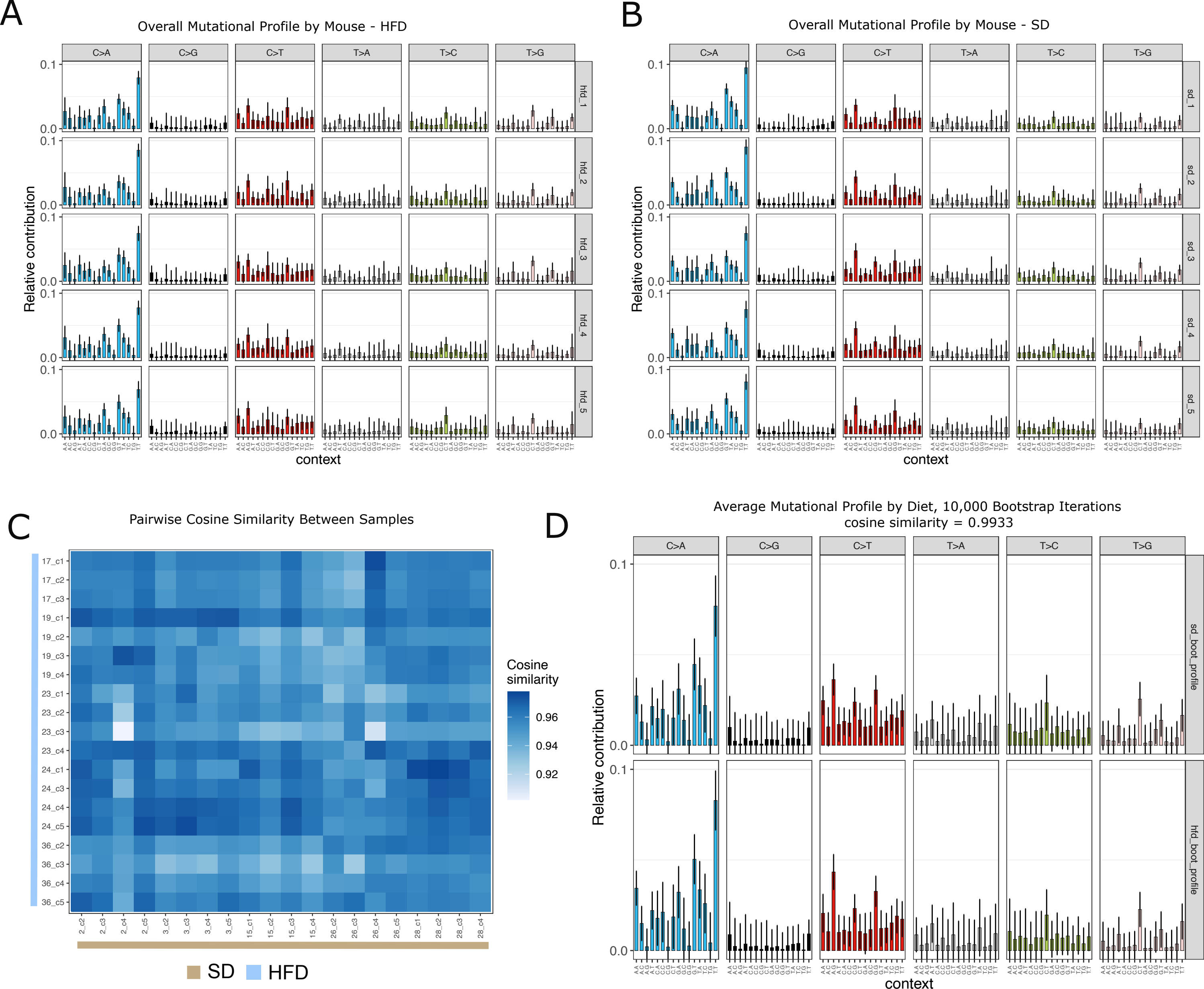

A
Overall Mutational Profile by Mouse - HFD
C>A
C>G
C>T
T>A
T>C
T>G
hfd_1
hfd_2
hfd_3
hfd_4
hfd_5
Relative contribution
context
B
Overall Mutational Profile by Mouse - SD
sd_1
sd_2
sd_3
sd_4
sd_5
C
Pairwise Cosine Similarity Between Samples
17_c1
17_c2
17_c3
19_c1
19_c2
19_c3
19_c4
23_c1
23_c2
23_c3
23_c4
24_c1
24_c3
24_c4
24_c5
36_c2
36_c3
36_c4
36_c5
2_c2
2_c3
2_c4
2_c5
3_c2
3_c3
3_c4
3_c5
15_c1
15_c2
15_c3
15_c4
26_c2
26_c3
26_c4
26_c5
28_c1
28_c2
28_c3
28_c4
Cosine similarity
0.96
0.94
0.92
SD
HFD
D
Average Mutational Profile by Diet, 10,000 Bootstrap Iterations
cosine similarity = 0.9933
sd_boot_profile
hfd_boot_profile

Supplementary Figure 2. (A) Average 96-channel SNV mutational profile per mouse in the HFD group. Error bars indicate ±1 standard deviation from the mean. **(B)** Average 96-channel SNV mutational profile per mouse in the SD group. Error bars indicate ±1 standard deviation from the mean. **(C)** Pairwise cosine similarity matrix between mutational profiles of all clones from SD and HFD mice respectively. The color scale has been adjusted to reflect the range of represented values from 0.9 to 1.0. **(D)** Aggregated mutational profiles by mean for each diet group after 10,000 bootstrap iterations of the mutational matrix of SD and HFD clones respectively. Error bars indicate ±1 standard deviation from the mean. The reported cosine similarity between the two averaged bootstrapped profiles is 0.9933.

A

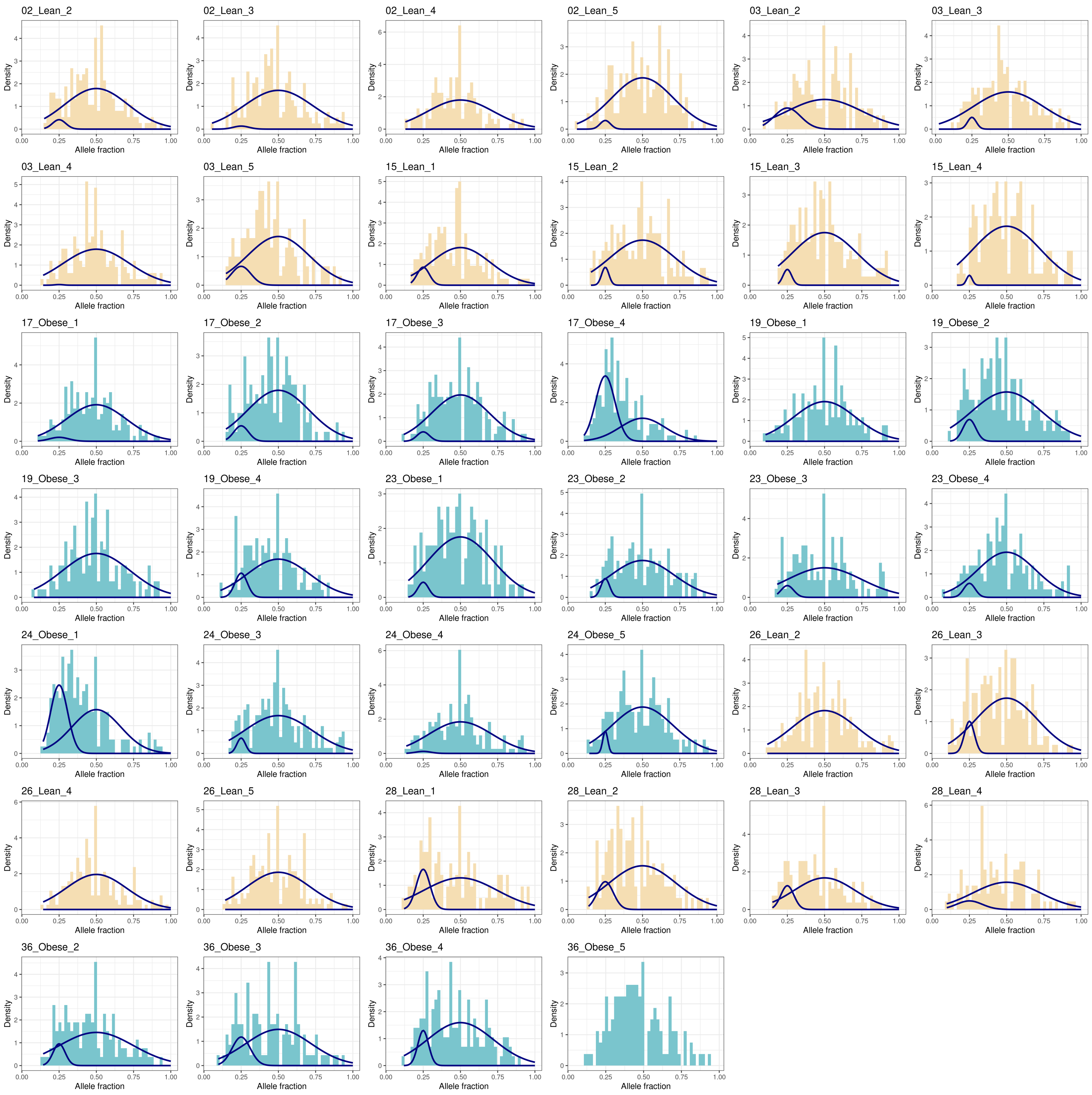
02_Lean_2
02_Lean_3
02_Lean_4
02_Lean_5
03_Lean_2
03_Lean_3
03_Lean_4
03_Lean_5
15_Lean_1
15_Lean_2
15_Lean_3
15_Lean_4
17_Obese_1
17_Obese_2
17_Obese_3
17_Obese_4
19_Obese_1
19_Obese_2
19_Obese_3
19_Obese_4
23_Obese_1
23_Obese_2
23_Obese_3
23_Obese_4
24_Obese_1
24_Obese_3
24_Obese_4
24_Obese_5
26_Lean_2
26_Lean_3
26_Lean_4
26_Lean_5
28_Lean_1
28_Lean_2
28_Lean_3
28_Lean_4
36_Obese_2
36_Obese_3
36_Obese_4
36_Obese_5
Density
Allele fraction

Supplementary Figure 3. (A) Variant allele frequency distribution (VAF) of insertions and deletions (indels) for each organoid clone modeled with a Gaussian distribution, after deduction of germline variants found in the mouse tail sequences. Gaussian mixture model was fit with fixed means at 0.25 and 0.5 to identify the proportions of clonal and sub-clonal cell populations. The resulting distributions are shown in blue.

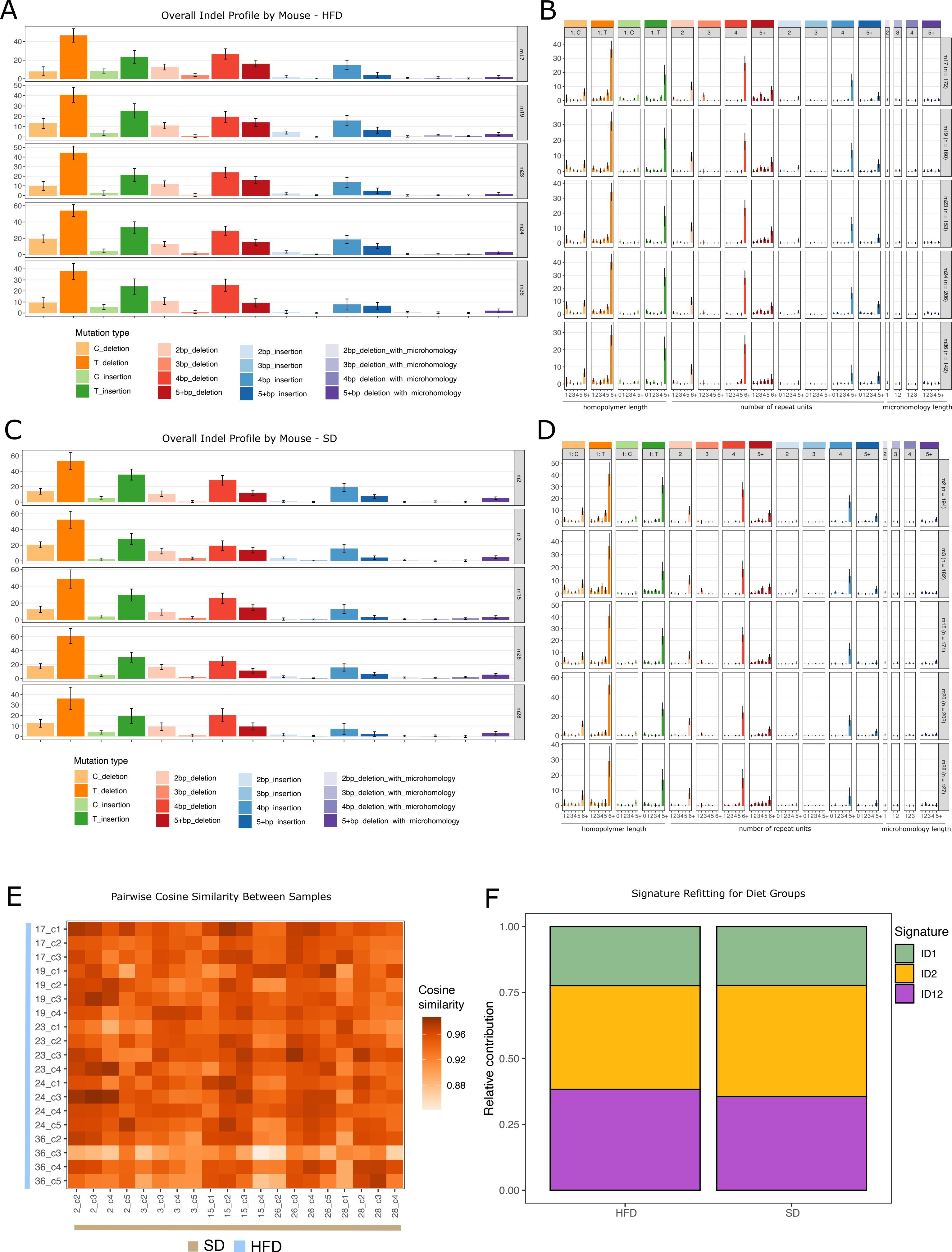
A
Overall Indel Profile by Mouse - HFD
m17
m19
m23
m24
m36
Mutation type
C_deletion
T_deletion
C_insertion
T_insertion
2bp_deletion
3bp_deletion
4bp_deletion
5+bp_deletion
2bp_insertion
3bp_insertion
4bp_insertion
5+bp_insertion
2bp_deletion_with_microhomology
3bp_deletion_with_microhomology
4bp_deletion_with_microhomology
5+bp_deletion_with_microhomology
B
homopolymer length
number of repeat units
microhomology length
C
Overall Indel Profile by Mouse - SD
m2
m3
m15
m26
m28
Mutation type
C_deletion
T_deletion
C_insertion
T_insertion
2bp_deletion
3bp_deletion
4bp_deletion
5+bp_deletion
2bp_insertion
3bp_insertion
4bp_insertion
5+bp_insertion
2bp_deletion_with_microhomology
3bp_deletion_with_microhomology
4bp_deletion_with_microhomology
5+bp_deletion_with_microhomology
D
homopolymer length
number of repeat units
microhomology length
E
Pairwise Cosine Similarity Between Samples
Cosine similarity
0.96
0.92
0.88
17_c1
17_c2
17_c3
19_c1
19_c2
19_c3
19_c4
23_c1
23_c2
23_c3
23_c4
24_c1
24_c3
24_c4
24_c5
36_c2
36_c3
36_c4
36_c5
2_c2
2_c3
2_c4
2_c5
3_c2
3_c3
3_c4
3_c5
15_c1
15_c2
15_c3
15_c4
26_c2
26_c3
26_c4
26_c5
28_c1
28_c2
28_c3
28_c4
SD
HFD
F
Signature Refitting for Diet Groups
Relative contribution
1.00
0.75
0.50
0.25
0.00
HFD
SD
Signature
ID1
ID2
ID12

Supplementary Figure 4. (A) Average 16-channel indel mutational profile (main indel contexts) per mouse in the HFD group. Error bars indicate ±1 standard deviation from the mean. **(B)** Average 83-channel indel mutational profile (extended indel contexts) per mouse in the HFD group. Error bars indicate ±1 standard deviation from the mean. **(C)** Average 16-channel indel mutational profile (main indel contexts) per mouse in the SD group. Error bars indicate ±1 standard deviation from the mean. **(D)** Average 83-channel indel mutational profile (extended indel contexts) per mouse in the SD group. Error bars indicate ±1 standard deviation from the mean. **(E)** Pairwise cosine similarity matrix between indel mutational profiles of all organoid clones from SD and HFD mice. The color scale has been adjusted to reflect the range of represented values from 0.84 to 1.0. **(F)** Best subset refitting of diet groups to known indel signatures. The relative contribution per diet group is shown.

A

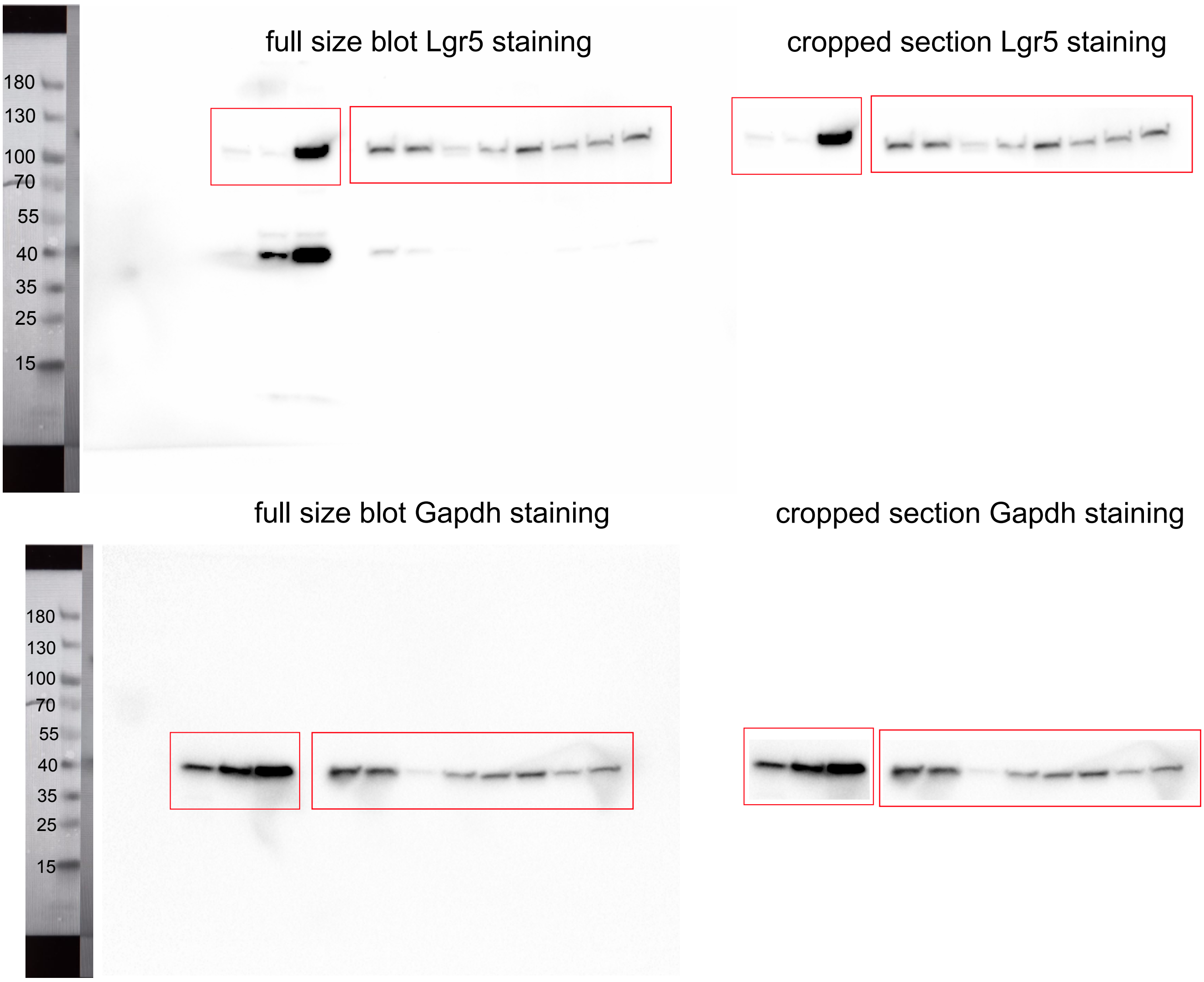
full size blot Lgr5 staining
cropped section Lgr5 staining
180
130
100
70
55
40
35
25
15
full size blot Gapdh staining
cropped section Gapdh staining
180
130
100
70
55
40
35
25
15

Supplementary Figure 5. (A) Original uncropped gels for the Western Blot presented in Supplementary Figure 1. The top row shows the staining with the Lgr5 antibody (full sized gel on the left, cropped section to the right and marked in the red squares). The bottom row shows the staining with the Gapdh antibody (full sized gel on the left, cropped section to the right and marked in the red squares). Molecular size marker (ladder) as imaged by chemiluminescence using the Chemidoc XRS+ imaging system (BioRad) is aligned to the final blot.
